# Extra Supplement—The Nursing Mirror

**Published:** 1889-04-13

**Authors:** 


### The Nursing Mirror.

All Contributions for this Supplement should be addressed "The Nursing Mirror," care of the Editor, 139-140, Salisbury Court, Fleet Street, London, E.C.

### En Passant.

**LADY NURSES.**—Though the correspondence in the *Standard* on this subject has ceased, it has been taken up in some of the local papers, and most extraordinary articles are appearing on nurses and nursing. A Shrewsbury paper has discovered that five years' probation is too long, and we quite agree with it. Again, it is proposed that a philanthropic body of ladies should take up the subject and alter the rules of the hospitals. Seeing that in these days it is trained nurses who are generally appointed matrons—a fact which would not apply to a few years ago—we think the remedy is in the hands of the nurses themselves. Only we would beg every nurse as she rises in her profession to keep a vivid remembrance of her probationer days. By the time a woman becomes a matron she is often liable to have forgotten her first professional efforts; all her thoughts are centred on good management and strict economy such as shall win the praise of the committee. A matron's life is a busy one; her mind has to ponder many subjects, and those far-off probationer days fade out of her memory. This is why there are still a few hospitals where lady nurses have cause to grumble, but certainly they are very few.

**WOMEN AND DRESS.**—Mr. J. W. Taylor, of Birmingham, has written an article for the "Medical Annual" on the "Diseases of Women in Relation to Health." This is a subject on which the sensible conversation of a nurse may be of great benefit to the patient; for the nurse generally knows whether a patient is healthily clad or not. The evils of tight lacing are both direct and indirect, and their reason is often hard to discover. A nurse can notice such a point better than a doctor; she can also speak more fully, and probably illustrate her remonstrances with graphic illustrations. She can also show practically how corsets can be replaced, for with many women it is useless and unwise to ask them to forswear stays. It is all very well for a doctor to say a woman is not to wear corsets, but if he realised the weight of her skirts, he would know that some substitute must be found to keep pressure off the waist. A nurse can devise a bodice such as young children wear, to which garments can be buttoned. Mr. Taylor numbers relapsing anæmia and many horrible illnesses amongst the effects of tight lacing, but he is wise enough to see that moderate support is necessary in the cases of most women, and contributes to the ability for active work.

**MISSIONARIES AND MASSAGE IN INDIA.**—The prize day of Hyderabad Medical School was held on January 25 last, and the principal, Dr. Lawrie, made some remarks about massage and medical missionaries, which are exceedingly interesting. Dr. Lawrie is a great friend to medical women, and when ill lately was professionally attended by Dr. Dora White, one of the lecturers to the Hyderabad Medical School. But though Dr. Lawrie has the utmost faith in fully qualified women, he contends that there can be no greater mistake than the present tendency to allow partially-educated women to practise medicine in the zenanas. With regard to massage, Dr. Lawrie says that no people in the world are cleverer at massage and shampooing than the native *dais* and bearers, and that a lady doctor would do well to make herself familiar with their plans of treatment. There are eight ladies now studying medicine at Hyderabad, and Miss Furdonji, a young Parsi lady, lately passed her examinations with such credit that Lady Dufferin awarded her a special medal. The Nizam of Hyderabad, Sir Asman Jah, has also introduced scientific nursing into the Afzul Gunj Hospital, and nurses are being trained there under Miss Thornton.

**PAUPER NURSES.**—The York Board of Guardians has spent £900 on the erection of a workhouse fever hospital, and has now arranged with the Urban Sanitary Authority to take fever cases at the city hospital at a charge of 10s. 6d. a week. Why not use their own hospital? our readers naturally ask. Because, in their own words, "It could not be supposed that the House Committee would for a moment sanction the nursing in that hospital of patients by paupers who were wanting in every essential requisite of a nurse." But surely this is not going to the root of the matter; would it not be simpler to provide proper nurses? We sincerely hope that the coming elections will place many more lady guardians on the different Boards, and that through their influence every union infirmary will shortly be scientifically nursed.

**NATIONAL PENSION FUND FOR NURSES.**—The Governors of Guy's Hospital have determined to affiliate that institution to this fund by paying one-half of the premium of each of their sisters and nurses. This decision has not been arrived at without full deliberation, and it is felt that it will be widely followed, especially by hospitals who have nursing institutions attached to them. The scheme is the joint work of Mr. E. H. Lushington, the Treasurer, and Dr. Steele, the Medical Superintendent. At a meeting of the council held on the 4th inst., Mr. Walter H. Burns in the chair, the honorary manager reported that the number of applications for pensions and sick pay up to date was 777 (exclusive of the Mildmay nurses, St. John the Divine nurses, and the nurses of the Seamen's Hospital), of whom 623 had paid their contributions, amounting to £12,300, of which £1,524 had been received since the last monthly meeting; 52 applications had been received during the corresponding period, and 44 were accepted at the present meeting. It was reported that the funds invested amounted to upwards of £35,800.

"**A FELLOW FEELING.**"—With regard to the combination of private nurses there is one fact which must be considered, that women are somewhat lacking in the fellow-feeling which men, especially young men, so eminently possess. Dr. Richardson, of Boston, expressed this well when he said: "Nurses should always consider themselves as members of a grand sisterhood, and be ever ready to defend each other; for one never knows when they themselves may need defence. Nothing has struck me more, in my connection with this training school and the Harvard Medical School, than the striking difference between the students of the two institutions. In the medical school there is a spirit of clanship and good-fellowship, and for a student—to use a popular phrase—to 'go back on' another student is almost unheard of; while here, and especially in private practice, it is rare to find a nurse who will shield another who had made a mistake." A little more tolerance and a little less jealousy, a little more fellow-feeling and a little less reserve, would forward the interests of nurses as much as holding meetings or writing letters. Who has not seen one nurse get into the same railway-carriage with another, and though the two will eye one another furtively, they will never speak. But if one soldier is thrown into contact with another, the mere uniform is enough to produce a feeling of good-fellowship, and one will cry, "Hullo, comrade! where are you going?" But if, on the other hand, a doctor says to a nurse at a private case that she is looking overworked, and he must send for a second nurse, the usual answer is, "Oh, doctor, I'd rather manage by myself." Instead of a free and frank friendship amongst these fellow-workers, there is dislike and suspicion, and the very bonds which one would think would link these women together are made the cause of jealousy and estrangement. Those nurses who are going to combine together for the advantage of all, had better remember that a "fellow feeling makes us wondrous kind."

### Nursing in the Soudan.

### Part I.—The Start from England.

*(Continued from page iii.)*

A large part of the square and streets of Alexandria were still in ruins, though the best was being done to rebuild them. Except in the European quarter, there was the same pitiable amount of dirt, squalor, and wretchedness to be seen as in most other Eastern cities; although the warmth and glorious sunshine throw a glamour over it, and seem to make up for the lack of much that we, at home, know as comfort. A drive in the city, past the Mahmoudieh Canal, to Pompey's pillar, and through the many narrow, irregular street and bazaars to the quay, brought us once more to our ship: we settled down for another steam of some 12 hours to Port Said, where we were allowed to disembark for a few hours while coaling took place. There was very little of interest at Port Said, which resembles a French seaport town, with no beauty of its own, and nothing to see. The sisters in their pretty uniform excited a good deal of interest among the natives, who watched them about with curious eyes. Steaming along the canal for some hours, the last of eight ships in a line, we saw all sorts of unusual sights; strings of camels, with their picturesque Bedouin drivers, a mirage, large flocks of flamingos, pelicans, and other strange birds. The heat was oppressive in the narrow channel of water meandering through the desert. At equal distances there are sidings in which ships anchor to allow others to pass, and the close proximity of a strange vessel caused some little excitement. We scanned our fellow-travellers with keen interest, and saluted them by dipping our flag. We passed Ismalia, a pretty, bright place with some vegetation, which is a novelty in this part of the East. One morning, when carefully steaming along our narrow channel, the ship in front of us stuck fast, and we, in trying to avoid collision, stuck too. This was an amusing *contretemps*. Out jumped the sailors, and by vigorous pulling of the hawsers, and still more vigorous use of their lungs, we were righted, and anchored for the night.

### Part II.—Going into Action.

Three or four days after leaving Alexandria we sighted Suez, the end, as we fondly hoped, of the long and tedious voyage, but there is no accounting for the exigencies of war. On coming to an anchor, the embarkation officer came on board, and said a telegram had been received from Suakim that morning, stating that the enemy had surprised the troops, that several of our men had been killed and many wounded, and asking them "to send all available sisters down immediately."

In military life obedience is the first duty, and orders must be obeyed; so, putting our chagrin aside, we disembarked, bag and baggage, and took up our new quarters in the troopship "Queen" for a three days' steam down the Red Sea to Suakim. At Suez two of the National Aid Society's sisters received orders to take train for Cairo for work up the Nile. Good-byes were said, and they left us after three weeks' pleasant intercourse. Our party was, however, reinforced by the addition of four sisters who joined us *en route* for Suakim.

We had on board many camels and mules, and a company of marines, with their officers. Land was seen very clearly as we steamed down the Red Sea, and the colouring was indescribably beautiful. The heat was far greater than any we had experienced, the thermometer, although there was a stiff breeze blowing, standing at 80deg. It would have been pleasant enough had it not been for the insect creation, which drove us to desperation—flies, fleas, mosquitos, and worse! At last, however, we reached Suakim, with its flat-roofed houses, Arab huts, low hills in the distance, and endless stretch of desert behind, without a single tree to be seen. We had a good view of our own camp on the right, with its white tents occupying a large area of ground. Upon the left all eyes quickly became concentrated, for through our field-glasses we could clearly make out an engagement. The enemy had attacked a convoy sent out to reconnoitre, and our men were to be seen in the square formation, the enemy, apparently, attacking them simultaneously from all sides. In our extreme excitement we seemed, for the moment, to forget what the terrible reality meant to our poor fellow-creatures. The officers and marines on board bewailed their hard fate that they were not in time to help their comrades.

The pilot presently came out to us, and we found, to our disappointment, that there was no chance of entering the harbour for some hours. The approach is dangerous, except at high tide, and ships are not allowed to run the risk, so there was nothing for it but to steam away and anchor some miles off, with as good a grace as we could, until the signal from land was given to tell us we might safely enter the harbour. Earnestly was that signal watched for, and when it came no time was lost in weighing the anchor, and making ready for the start which was to bring us to the scene of our nursing labours. None of us would be sorry to be at work, for pleasant as a sea voyage is to most, there comes a time when work is pleasanter, and we were eager to render what help we could to the poor fellows who had taken their part so nobly in that cruel war. The harbour, which is a capital one, due to the natural formation of the land, presented a novel appearance. Huge troopships, smart gunboats, torpedo-boats, and native craft of the most primitive description, from a respectable sailing boat to the simple trunk of a tree hollowed out, were all mixed up in that un-English looking harbour.

Our orders from headquarters were to remain on board until morning, when we should receive directions as to our future work. We therefore amused ourselves with watching the many curious scenes around us. One of the oddest things imaginable was the disembarkation of our camels. Could anything be more comic than the desperate attempts these ungainly animals made to escape from the clutches of those whose business it was to hoist them up in slings, and swing them over on to large flat-bottomed boats which were alongside waiting to take them to land? In the morning we patiently waited for the principal medical officer to come on board, speculating, as best we could, upon our possible location in the future. When he arrived, it is difficult to convey to my readers our complete dismay upon finding that the work destined for the acting superintendent and three sisters, as soon as arrangements could be made, was to nurse the sick and wounded officers and men about to embark for England on board the troopship "Iberia." In spite of the interest in the nature of the work, gruesome visions would arise of the horrors of sea-sickness so lately experienced, discomforts of a long sea voyage, and the cooped-up nature of our field of action.

However, we had not come all the way from home to choose our own work, or to grumble at that assigned to us, so we pulled ourselves together and cheerfully accepted the task committed to us. We could not start immediately, as the vessel in which we were destined to take our voyage was not yet in port, being on her way from Australia with the gallant fellows who were coming from that distant colony to help their brothers of the mother country.

Three sisters of H.M. Nursing Service, who joined us at Suez, were told off to the base hospital at the H Redoubt, while one was to join the hospital ship "Ganges." The two others of our immediate party were instructed to organise the auxiliary hospital on the island, which is joined to the mainland by the causeway made some years ago by General Gordon. Whilst awaiting the troopship, the acting-superintendent and sisters rendered what help they could in nursing the sick on board the hospital ship "Ganges" (permanently anchored in the harbour), and to the two sisters who were organising the auxiliary hospital. Their needs were, for the time, the most pressing.

*(To be continued.)*

### The Nursing and Management of the Insane.

By T. Duncan Greenlees, M.B. Edin.

*(Being the introductory to a course of lectures to the Nurses of the City of London Lunatic Asylum.)*

*(Continued from page ii.)*

A trained nurse can go through more work than an untrained one; and this work is done with less expenditure of energy, is performed with greater pleasure, and the result itself is always found to be more satisfactory. A discussion in the daily papers some time ago on "What to do with our daughters," suggests to me that there is a great demand in asylums for educated women, and that were "our daughters" to overcome the unreasonable repugnance many of them have to asylum life, nursing the insane, and attending to their wants would be a much more sensible way of devoting their lives than waiting for the fabled prince, who tarries long!

In every well-regulated asylum, as in a general hospital, there are wards set apart for the nursing of those patients suffering from some form of bodily, as well as mental, disorder, and the cheerfulness of your sick patients' surroundings should always be a point of paramount importance to you. Any person of the nature of Job's comforters should be religiously kept out of the way, and those of you in attendance should possess cheerful and pleasant manners, and, without being ostentatious, give the patient the impression of your being a reliable and cheerful support to her in her affliction. Among the insane very frequently the only apparent sign of illness is loss of appetite; you should, therefore, in every case where the patient refuses her food, direct the doctor's attention to her.

Of course, such symptoms as cough, shortness of breath, excessive thirst, spitting of blood, diarrhœa, or faintness, will be too obvious to be overlooked, but it is well to remember that little and apparently trifling symptoms are too frequently the first and only indications we find of commencing serious disease, and they accordingly demand our earliest attention. To do this successfully you should make yourselves thoroughly acquainted with the habits of everyone under your care; you will then be able to note at once any slight deviation from health. Prompt attention on your part is an important matter, for many insane patients never complain when they are really ill, and, in some cases, mental disease has such an effect upon the system that your patients may suffer great pain and yet refrain from complaining to anyone about it.

The strictest attention s ould be given to t e directions of the doctor as to the administration of food and medicines, both as regards the time of giving them and the quantities to be administered. A patient ought not to be given anything simply because he asks for it, and fruit, etc., brought by friends of the patients must not be given until permission is obtained. Disobedience to this rule, in certain cases of illness, has been known to result in the death of the patient. You should educate yourselves to observe carefully, so that, when questioned by the medical officer regarding the sick under your care, you will be able to give the result of your observations in an accurate and succinct manner; the more concise your information is the greater will be its value.

Those patients who can only take their food in a fluid condition should have it administered frequently and in small quantities. It should be served in a clean and tempting way, for a sick patient's appetite is capricious, and requires much humouring; such a patient may refuse food, even although his appetite may be good, owing to the careless and slovenly manner in which it is served to him.

There are other rules and instructions referring to the nursing and care of the sick insane, which I shall refer to in a future lecture. We must now study some of the laws that govern asylums and their inmates, as well as those enactments having reference to your duties to the institution with which you are connected.

The management of all asylums is under the most stringent legal regulations, to which every official has to submit, and every certified insane person having been deprived of his liberty, and being legally irresponsible for his actions, is protected by the State, so that his person is taken care of and his affairs properly managed during his incarceration. In this philanthropic age large sums of money are annually expended to provide for the care of the insane—a class who used formerly to overcrowd our prisons and workhouses.

In one of the recent lunacy enactments, an attendant on the insane is legally defined as follows (Vict. 16 and 17, c. 96, sect. 36):

"An attendant shall mean any person, whether male or female, who shall be employed, either wholly or partially, in the personal care, control, or management of any lunatic in any registered hospital, or licensed house, or of any single patient."

The following remarks were made by the Commissioners in Lunacy, in one of their annual reports, relative to the duties of attendants and nurses on the insane:

"The skill and judgment of a superintendent . . . are of little avail unless he is zealously supported, and his orders effectually carried out by an adequate staff of well qualified attendants. To them, in a great measure, must of necessity be entrusted the personal charge of the patients. . . . Attendants should be firm, and at the same time combine gentleness with firmness, and they should be able to superintend, direct, and promote the employment and recreation of the patients."

*(To be continued.)*

### Work to Do.

Ladies seem to think that nursing is the only good work open to them, and that if they wish to help the sick poor, their only plan is to enter a hospital as probationers. This idea is erroneous; only a special love of nursing ought to urge a woman to become a probationer: mere sympathy with suffering is not enough. Sympathy is very useful when dealing with the sick, but in nursing them knowledge is more useful, and physical and moral strength are not to be despised. Again and again it has to be proved that every woman is not a born nurse, and we would urge on any who think of entering a hospital to consider whether they have chosen the right path, and if outside work will not give them all they want to do. The *London Diocesan Magazine* gives a list of visitors and helpers wanted every month, and here those who want work can find several ways in which their need can be supplied. Ladies are wanted as visitors in workhouses, infirmaries, and hospitals, where their duties will be to read to convalescents, amuse children, or sympathise with those who are in sore distress. Ladies are also wanted who will collect old linen and forward it to some of these medical charities. Again, there are numerous parishes where district visitors are wanted; also Sunday-school teachers. Helpers are needed for sewing and other classes, and for mothers' meetings and temperance meetings. One society needs a secretary, another a visitor who can talk French and German. Then there are appeals for permanent aid if ladies desire to devote their services to the poor for ever. A mission home at the East-end asks ladies to forsake their luxurious lives at the West-end, and come and live and work amongst the struggling poor.

There can be no doubt that those who want work to do can here find an outlet for their energy, and probably combine home duties with their wish to help the sick and needy. The present rush of unsuitable candidates into the nursing field is simply the result of this form of work being kept much before the public at present; and the equally good work done on other lines being partially forgotten in fashionable circles. But all should remember that nursing is a profession, the work of a lifetime, and is not to be lightly taken up, and then quickly dropped. The unmarried girl seeking temporary employment to pass the morning hours, could do more good as a reader to patients than as a nurse. The middle-aged woman, whose teachable years and strongest period are past, and who begins to think she should do some good before death overtakes her, will find her age and experience more useful in a mission home than in a hospital. The long hours and strict discipline of a probationer's work are not suitable for those who have crossed the line of middle age. Nursing needs learning between twenty and thirty, practising from thirty to forty, and then till sixty the post of matron is desirable.

It is a strong proof of the great good existing in all women's hearts that so many of them desire the life of self-denial offered them as nurses; but it is pitiful to see a lady take up a work for which she has neither strength nor time, when suitable women are waiting for the post she holds, and other lighter work is crying for supporters. In this one magazine alone numerous workers are wanted in various branches; there must be much good left undone throughout the country simply because "doing good" is more fashionable just now in cap and apron than without them. To ask in vain for readers and visitors for hospitals, to have the lighter but not less important work left, while all try to grasp the most difficult task, is not commendable to women's usefulness and modesty. Let some of us who are not nurses acknowledge we are not fitted to become so, and lend our aid to diocesan work instead.

### Everybody's Opinion.

*Correspondence on all subjects is invited. No communications can be entertained if the name and address of the correspondent is not given, or unless one side of the paper only be written on.*]

### THE PENSION FUND.

NURSE ELIZABETH writes: I see in this week's *Hospital*, in a letter from "Handclasp," she considers that "The National Pension Fund has all it can do to scrape together the required 1,000 members." Perhaps it has at present, but, perhaps, there are some hundred nurses, like myself, waiting for the 1st January, 1890, to see if the required 1,000 have joined, and, if so, to join themselves. I approve of the Pension Fund—in fact, I consider it a grand thing, and heartily thank all those gentlemen who have shown their great interest in nurses by bounteous goodness and generosity. I think nurses would not be so backward in joining if the pensions were given at 50 instead of 60 years of age. I doubt whether in this generation you will find many nurses able to do much work after the age of 50. If there are any, by all means let them work as long as they are able, but I think a good plan would be to give a nurse her pension at 50 years of age, or as soon after as a medical man considered it necessary for her to give up work (it might be a doctor chosen by the Committee of the National Pension Fund if thought desirable by them). Now, suppose pensions were given at 50 years of age, £15, £22 10s., or £30, according to our contributions, you could not, of course, afford to give bonus additions later on, nor should we expect it. I have also thought of another plan. I know of several nurses standing quite alone in the world, without a relation, and I daresay there are many others like them. Don't you think it would be a good thing to build a "Home," and install a matron and trained nurses and servants for such poor friendless nurses. In such a home, where they had every thing they required, from a bed to lie upon to medical attendance and trained nursing when they were sick, they would not require a pension. I feel sure there are many who would prefer that to ekeing out a small pension in lodgings among strangers. In a home of that kind nurses might have a very happy old age, and how interesting for several nurses to get together in the "general sitting-room" and talk over "old times."

### NURSING THE INSANE.

A TRAINED NURSE writes: As a constant reader of *The Hospital*, and matron at present of a small staff of asylum nurses, may I be allowed to say a few words on "Nursing the Insane"? The idea that trained hospital nurses do not make good asylum nurses I hope will be short lived. In my opinion (and I speak from experience) nursing the insane should take a place in the complete hospital training of a nurse. I would suggest, if we are to have our asylum nurses more patient, gentle, and intelligent in their dealings with their patients, place at the head of them for their matron and head attendant hospital-trained nurses, who will instruct them by classes and diagrams. There is no royal road to general nursing. Ventilation, feeding patients, bed-making, taking of temperatures, making poultices, treatment of bed sores and epileptics, are the same for the insane as the sane, only no doubt with the former more difficulty will be expected. With my small staff we have weekly classes, illustrated by the help of a blackboard, which my committee kindly granted, and it more than repays one to hear how delighted the nurses are with the knowledge they acquire. To illustrate a fractured tibia, I had a doll and bandaged the limb, fully explaining all particulars even to the making of a fracture bed and changing of sheets. Insane patients sometimes fracture their limbs, is it not necessary to teach the asylum nurse how to treat it? Also I have a regular Bible class with my nurses, and they tell me how they quite enjoy the lull from their busy life. If we would only give our asylum nurses the same privileges in the way of instruction we do our probationers, we should soon have a higher tone among them. I do not consider the question of increasing their salaries has anything to do with the quality of nurse to be engaged. Our night, charge, and second-class nurses are as well paid as any hospital staff nurse, for in most asylums they have their uniform given and made, also collars, cuffs, belts, hats, and shawls are included in uniform. Do away with the theatrical performances and fancy dress balls for our asylum nurses, then we shall have more interest taken in the patients, and more thoroughness in the ward work. If balls are necessary for the nurse's recreation, let them be in uniform, and let the nurse's only anxiety be "Have I a clean apron, collar, cuffs, and cap for the ball to-night?" Also shorten the time of these balls. It is not possible for a nurse who has been on duty for 13 hours during the day to be dancing up to 11 p.m., or, what now and then happens, until 4 a.m., to be able to do her duty at all the next day, or to be patient with those committed to her care.

E. T. writes: I am indeed glad to learn you are about to devote more of your space to asylums. Unlike your correspondents who accuse you of neglecting our interests, I feel sure the fault lies with ourselves. After having read from the first your long-needed journal, I may endorse my humble opinion that each and all will receive impartial treatment if they do but go to you with anything worth consideration, and so I take courage to give you my small growl, with the hope that abler pens may take up my plea or show me I am wrong. That asylum attendants are not made much of goes without saying; and still no work, if well done, is more useful. If people show disregard for the servants, why carry their neglect unto the patients? As an officer of some years' experience, I can say (with one exception) it has never been my pleasing duty to take to our hospital ward (for the insane are sometimes physically sick) a gift of a bunch of flowers, a little fruit, a few pictures, or some game. Such might well be afforded, and not missed, by the gentry in the neighbourhood, or by the wives and daughters of our visiting committees. I almost fancy I hear it said, "Oh! it is all too painful; I could not bear to see those poor creatures." A parcel addressed to the medical superintendent would be placed in the hands of a responsible officer, who would see the gift judiciously divided. No need to brave the sight of anything painful. And yet, if the thought of the insane is so repellent, it would seem but charitable to bestow not only encouragement, but some thought and feeling on those who, by reason of gaining a livelihood, are obliged to devote their constant attention to cases who may be particularly interesting, but are at all times unreliable, sometimes dangerous, and often unsightly. How nice it would be if some kindly-disposed lady threw open her doors and gave the overlooked attendant a glimpse of refinement and pleasure—music, bright readings, or a picnic in her grounds—by offering them the means of relaxation, and showing by encouragement that her sympathies were with a most useful class. She would set the example to others, and would raise and help the attendants to gain that refinement which at present they are supposed to lack. You will be pleased to know your instructive and interesting journal is largely distributed throughout this institution.

### Vacancies.

The following vacancies are announced:

**Matron and Lady Superintendent.**

Bradford Infirmary, £80.

**Nurses.**

Bristol Hospital for Children and Women.
City Hospital, Liverpool, £25 and £35.
Royal Hospital, City Road, £22.
Workhouse Infirmary Nursing Association.
Newcastle-on-Tyne, £25.
Nurses' Institute, Weston-super-Mare.
Deaconess House, Chester, £24.
Eastbourne Nursing Institution.
Kingston Home, Hull.
Swansea Nursing Institute, £25.
Cancer Hospital, Liverpool.
Nurses' Institute, Leicester.
Skin Hospital, Liverpool.
Nursing Institution, 96, 97, 98*a*, Wimpole-street, London, W., Mr. Wilson has several vacancies for thoroughly-trained Nurses.
West Kensington Nursing Home.
Swansea Hospital, £20
Moor-Edge Hospital for Sick Children, Newcastle, £18.
Hospital for Consumption, Brompton (several).
Royal Albert Hospital, Devonport (several), £25.
Victoria Hospital, Hull.
Levick Institute, Paris (several).
Nursing Institution, Jersey, £25

**District Nurses.**

Nurses' Home, Frome, £20.
Stamford.
Hampton Wick, £60.
Corbridge-on-Tyne, £75.

**Probationers.**

Kent Nursing Institution.
Gateshead.
Blackheath, £24 premium.
Stoke-on-Trent.
Carmarthen, £8.

**Sisters.**

Infirmary, Bolton, £25.

Monsall Fever Hospital, Manchester, £30.

**Midwives.**

Chelsea Dispensary.

**Wardmaids.**

Borough Hospital, Halifax.

Cottage Hospital, Coltishall, Norwich.

### Notes and Queries.

**Queries.**

(4) *Printers of Certificates.*—Can anyone give me the address of a printer of certificates for nurses? *Matron.*

**Answers.**

*Belfast.*—Fiji is address enough. Try Kimberley, South Africa. We know of no hospital in Denver.

### Songs of Golden Deeds.

To dwell with evil loathed and drear,
High ladies leave their natal sphere,
To dwell where seeketh manifold offence
For delicate, well-nurtured sense,
Dividing holy heritage
Of inward treasure; there to wage
Deadly feud with the grim host
Of Satan, sharing all with lost
Wanderers in our wilderness,
Sallying to save and bless;
Yea, very bread of their own mind and heart
They break, celestial manna to impart,
Not hoard for mere "salvation"; gifts more blest
Than gold; faith, hope, and sympathy, with rest,
Strength, courage, wisdom, righteousness, and love;
They bring to earth health, healing from above.

*From* "A MODERN FAUST," *by the Hon. Roden Noel.*

